# Initial experience with the convergent procedure for longstanding persistent atrial fibrillation: A 5 year dataset

**DOI:** 10.1016/j.dib.2020.105417

**Published:** 2020-03-12

**Authors:** E. Maclean, J. Yap, B. Saberwal, S. Kolvekar, W. Lim, N. Wijesuriya, N. Papageorgiou, G. Dhillon, R.J. Hunter, M. Lowe, P. Lambiase, A. Chow, H. Abbas, R. Schilling, E. Rowland, S. Ahsan

**Affiliations:** a Barts Heart Centre, St Bartholomew's Hospital, W Smithfield, London EC1A 7BE, UK; b William Harvey Research Institute, Charterhouse Square, Barts and the London School of Medicine and Dentistry, Queen Mary University of London, London EC1M 6BQ, United Kingdom

**Keywords:** The convergent procedure, Hybrid surgical ablation, Longstanding persistent atrial fibrillation

## Abstract

In patients with longstanding persistent atrial fibrillation (AF), outcomes from catheter ablation remain suboptimal. The convergent procedure combines minimally invasive surgical ablation with subsequent catheter ablation, and may contribute towards maintenance of sinus rhythm in this patient group. We performed the convergent procedure on 43 patients with longstanding persistent AF from 2013–2018. Patients underwent clinical review at 3, 6, and 12 months and thereafter as necessitated by their symptoms. Our dataset describes patients’ baseline characteristics and rhythm control protocols, as well as outcomes including arrhythmia recurrence, the need for antiarrhythmic drugs, requirement for repeat rhythm control procedures, and complications. These data provide a real world insight into the risks and benefits of the convergent procedure in patients with longstanding persistent AF.

Specifications table

**Table:** 

Subject	Cardiology and Cardiovascular Medicine
Specific subject area	Electrophysiology: the convergent procedure (hybrid surgical ablation) to treat longstanding persistent AF
Type of data	Tables of raw data Kaplan-Meier plot
How data were acquired	Data were extracted retrospectively from patient's electronic and hard-copy clinical records
Data format	Summated outcome data in Table 1
	Survival distribution (Kaplan-Meier plot) in Fig. 1
	Raw numerical and categorical baseline and follow-up data in supplementary Table S1 (see supplementary file)
Parameters for data collection	Baseline parameters: age, gender, hypertension (HTN), diabetes, ischaemic heart disease (previous percutaneous coronary intervention), current or previous smoking history, echocardiographic data, duration of atrial fibrillation, anti-arrhythmic drug use, anticoagulation strategy, previous ablation or cardioversion (DCCV), pacemaker, history of hypertrophic cardiomyopathy, symptomatic class according to EHRA and NYHA guidelines Catheter ablation data: number of ablations required, ablation lines delivered, complications Surgical ablation data: access used, number of ablation lesions delivered, complications Follow-up data: repeat echocardiographic data, repeat symptomatic data, rhythm data according to clinic electrocardiograms (ECGs), holter monitor examination and/or pacemaker interrogation
Description of data collection	Table 1: - Summated outcomes for all patients according to procedure reports and clinical records Fig. 1: - Kaplan-Meier plot showing arrhythmia-free survival over time for patients undergoing the convergent procedure (allowing for multiple catheter ablations and anti-arrhythmic drugs) Supplementary Table S1: - Procedural data were extracted from operation notes and procedure reports - Rhythm data were obtained from 72 h holter monitors, clinic ECGs and pacemaker interrogations where available - Echocardiographic data were extracted from the hospital's imaging database - Symptomatic data and medical co-morbidities were discerned from clinic visits and electronic medical records
Data source location	City/Town/Region: St Bartholomew's Hospital, London Country: UK
Data accessibility	With the article: - Table 1: within the article - Fig. 1: within the article - Table S1: supplementary file
Related research article	E Maclean, J Yap, B Saberwal, S Kolvekar, W Lim, N Wijesuriya, N Papageorgiou, G Dhillon, RJ Hunter, M Lowe, P Lambiase, A Chow, H Abbas, R Schilling, E Rowland, S Ahsan The convergent procedure versus catheter ablation alone in longstanding persistent atrial fibrillation: a single centre, propensity-matched cohort study International Journal of Cardiology *Vol. 303, p 49–53, March 15 2020,* DOI: https://doi.org/10.1016/j.ijcard.2019.10.053

## Value of the data

• These data evaluate an emerging hybrid ablation technique for treating atrial fibrillation (AF). No randomised controlled trial data has yet been published on this subject, and previous observational data demonstrate significant heterogeneity regarding procedural success and the incidence of complications [2,3]. Our results include outcomes for individuals with very longstanding persistent AF; there is currently only limited published data for this patient group.
• The convergent procedure requires ongoing validation as a potential rhythm control strategy. Our data examines procedural success both at 1 year post procedure and long term, and includes a breakdown of complications. A full description of the risks and the efficacy of the procedure can inform clinicians as to its suitability as a treatment option for their patients.
• Our observational data can be incorporated into other registries to potentiate more large-scale analyses of the utility of the convergent procedure. By publishing our raw data in its entirety on a per-patient basis, researchers also have access to specific data subgroups, such as those individuals with pacemakers, cardiomyopathy or severe systolic dysfunction.

## 1 Data

The dataset contains raw numerical and categorical data for all patients at the point of recruitment and during follow-up. This includes baseline characteristics such as age, gender and duration of AF, echocardiographic and symptomatic data, and history of previous rhythm control procedures. The presenting rhythm is documented for each follow-up visit, and the total follow-up duration listed in months. Anti-arrhythmic drug use is described according to class of action. The latest echocardiographic and symptomatic data is also listed, and procedural complications are itemised. These data are displayed in supplementary Table S1.

**Fig. 1:**
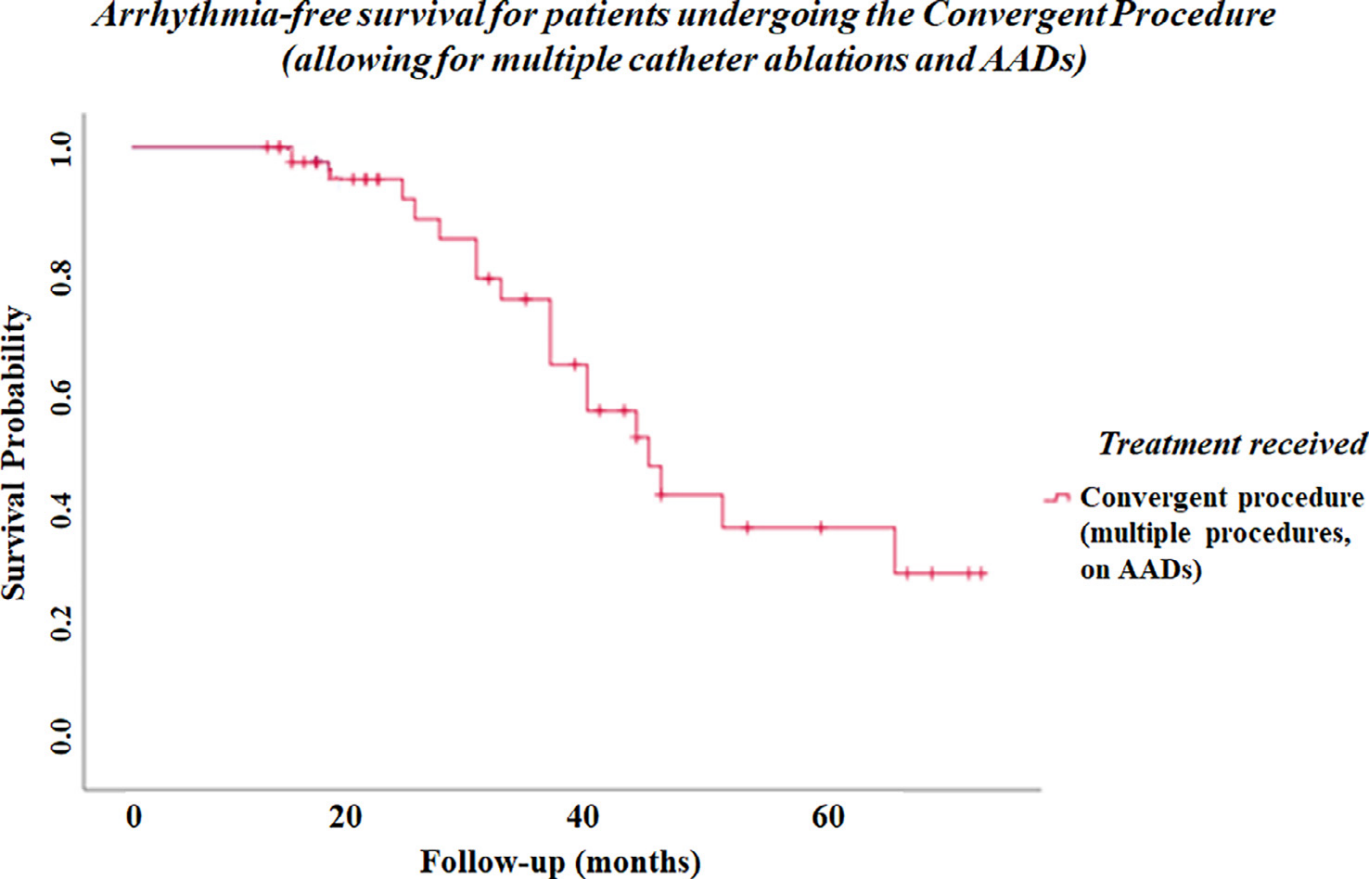
Kaplan-Meier plot showing arrhythmia-free survival over time for patients undergoing the convergent procedure (allowing for multiple catheter ablations and AADs).

Outcome data is summarised according to the primary and secondary outcomes, and is subdivided by duration of follow-up and the requirement for anti-arrhythmic drugs. Incidence of atrial tachycardia is also documented. These findings are described in Table 1, with long-term arrhythmia free survival visualised in Fig. 1.

**Table 1:** Clinical outcomes (at 1 year and long-term) for patients undergoing the convergent procedure.

Outcome	Patients undergoing the convergent procedure (n = 43)	
AF-free at 1 year (single procedure, on AADs)	**60.5% (n = 26)**	
AF-free at 1 year (single procedure, off AADs)	**37.2% (n = 16)**	
Arrhythmia-free long-term (multiple procedures, on AADs; mean follow-up 30.5±13.3 months)	**58.1% (n = 25)**	
Arrhythmia-free long-term (multiple procedures, off AADs; mean follow-up 30.5±13.3 months)	**32.5% (n = 14)**	
Off AADs at last follow-up	**60.5% (n = 26)**	
New atrial tachycardia	**32.6% n = 14)**	
Complications	**11.6% (n = 5)**	
	*Tamponade (pericardiocentesis)*	**2**
	*Emergency sternotomy*	**1**
	*Pericardial hernia*	**1**
	*Phrenic nerve palsy*	**1**

## 2 Experimental design, materials, and methods

### 2.1 Ethics

This project was registered with the local clinical effectiveness unit. As a retrospective analysis of patient records, the need for formal ethical approval was waived.

### 2.2 Methods

Our method for performing the convergent procedure has been described in detail elsewhere [1]. 43 patients underwent the procedure via a staged approach. Following the second part of the procedure (catheter ablation), a 3 month blanking period was observed in line with international consensus recommendations [4]. Subsequent clinical review took place at 3 months (with ECG), 6 months (with 72 h holter analysis) and 12 months (with echocardiogram, ECG and symptom-guided 72 h holter monitor). Patients with pacemakers in situ underwent device interrogation in addition to holter monitoring. Further clinical follow-up took place annually or sooner if warranted by symptoms.

### 2.3 Outcomes

A recurrence was defined as more than 30 s of documented AF outside of the blanking period. The presence of any other atrial arrhythmia was also recorded. Anti-arrhythmic drug (AAD) use was assessed at 12 months, and anticoagulation was continued as indicated by CHA_2_DS_2_VASc score. The primary outcome was AF-free survival at 12 months. Secondary outcomes included incidence of atrial tachycardia, change in NYHA and EHRA class, procedural complications, echocardiographic data, freedom from AADs, and arrhythmia-free survival long term. Patients underwent additional DCCV or repeat catheter ablation as indicated; in these cases, a further 3 month blanking period was observed and follow-up restarted.

## Conflict of Interest

The authors declare that they have no known competing financial interests or personal relationships that could have appeared to influence the work reported in this paper.

## Appendix Supplementary materials

Image, application 1
